# The effect of oxythioquinox exposure on normal human mammary epithelial cell gene expression: A microarray analysis study

**DOI:** 10.1186/1476-069X-3-9

**Published:** 2004-09-23

**Authors:** Maureen R Gwinn, Diana L Whipkey, Ainsley Weston

**Affiliations:** 1 Pathology and Physiology Research Branch, Health Effects Laboratory Division, National Institute for Occupational Safety and Health, Centers for Disease Control and Prevention, 1095 Willowdale Road, Mail Stop #L-2015, Morgantown, WV 26505-2888 USA; 2 Molecular Epidemiology Team, Toxicology and Molecular Biology Branch, Health Effects Laboratory Division, National Institute for Occupational Safety and Health, Centers for Disease Control and Prevention, 1095 Willowdale Road, Mail Stop #L-3014, Morgantown, WV 26505-2888 USA

## Abstract

**Background:**

Inter-individual variation in normal human mammary epithelial cells in response to oxythioquinox (OTQ) is reported. Gene expression signatures resulting from chemical exposures are generally created from analysis of exposures in rat, mouse or other genetically similar animal models, limiting information about inter-individual variations. This study focused on the effect of inter-individual variation in gene expression signatures.

**Methods:**

Gene expression was studied in primary normal human mammary epithelial cells (NHMECs) derived from four women undergoing reduction mammoplasty [Cooperative Human Tissue Network (National Cancer Institute and National Disease Research Interchange)]. Gene transcription in each cell strain was analyzed using high-density oligonucleotide DNA microarrays (HuGeneFL, Affymetrix™) and changes in the expression of selected genes were verified by real-time polymerase chain reaction at extended time points (ABI). DNA microarrays were hybridized to materials prepared from total RNA that was collected after OTQ treatment for 15, 60 and 120 min. RNA was harvested from the vehicle control (DMSO) at 120 min. The gene expression profile included all genes altered by at least a signal log ratio (SLR) of ± 0.6 and *p *value ≤ 0.05 in three of four cell strains analyzed.

**Results:**

RNA species were clustered in various patterns of expression highlighting genes with altered expression in one or more of the cell strains, including metabolic enzymes and transcription factors. Of the clustered RNA species, only 36 were found to be altered at one time point in three or more of the cell strains analyzed (13 up-regulated, 23 down-regulated). Cluster analysis examined the effects of OTQ on the cells with specific *p53 *polymorphisms. The two strains expressing the major variant of *p53 *had 83 common genes altered (35 increased, 48 decreased) at one or more time point by at least a 0.6 signal log ratio (SLR). The intermediate variant strains showed 105 common genes altered (80 increased, 25 decreased) in both strains.

**Conclusion:**

Differential changes in expression of these genes may yield biomarkers that provide insight into inter-individual variation in cancer risk. Further, specific individual patterns of gene expression may help to determine more susceptible populations.

## Background

Oxythioquinox (Morestan™ or OTQ, Bayer Corp) is a prototypical pesticide that was first used in 1968 on crops such as apples, pears, cucumbers, and gherkins. However, its use was later confined to non-food crops, limiting exposure to nursery and greenhouse employees. OTQ is a member of the quinoxaline class of pesticides, which also includes chlorquinox and thioquinox. Principal agricultural use of OTQ was limited to the states of California, Washington, Florida, New York, Pennsylvania, Ohio and Michigan [1]. The use of OTQ in the United States was voluntarily cancelled in 1999 with stocks in use until 2001, although OTQ is still listed in many different regions for use as an insecticide [2,3]. Further, OTQ is still in use today in other areas of the world, including Australia and the Caribbean [4,5].

Scientifically acceptable toxicity studies of OTQ are sparse. Early *in vivo *studies in rats found alterations of a variety of metabolic enzymes following OTQ exposures, including alkaline phosphatase [6]. Although not directly analyzed, the results of this study combined with an earlier study [7] suggest a direct effect of OTQ on succinate dehydrogenase, or other enzymes with a thiol group. Further work by Carlson et al (1970) [7] showed that, although OTQ has a low acute toxicity, cumulative exposure to this pesticide is not well-tolerated by exposed animals, with the majority of damage found in the liver of these animals. Further studies looking mainly at hepatic enzyme function found that OTQ exposure inhibits some hepatic enzyme functions [8]. OTQ was shown to be a carcinogen and hepatotoxin in laboratory animals in later studies [9]. OTQ has also been classified as a probable human carcinogen [10]. However, potentially carcinogenic exposures have already occurred and OTQ is still in use outside of the US, its mechanism of action remains of interest. In addition, OTQ has been shown to have an inhibitory effect on cytochome P450s, enzymes known to have a pivotal role in carcinogen metabolism [8,11-13]. Although early studies focused on hepatic effects, carcinogenic potential may occur in other tissues as well. While there have been multiple pesticides implicated in breast cancer, no studies have been published related to OTQ exposure and human cancer incidence [13].

This study is similar to work currently being carried out to determine gene expression profiles of a variety of environmental agents, including chemicals, physical agents and physiologic stresses [14-19]. The National Institute of Environmental Health Systems (NIEHS) has recently funded a consortium to expand the study of gene expression profile signatures for various chemicals, with the long-term goal to use these analyses in a validated gene expression profile signature database. A recent report by Shan etal. 2002 [20] is a good example of the differences between gene expression profile signatures for two chemicals in an animal system. This report profiled gene expression in rat carcinomas induced by two carcinogens, 2-amino-1-methyl-6-phenylimidazo [4,5-*b*]pyridine (PhIP) and 7,12-dimethylbenz [*a*]anthacene (DMBA), and was able to show that while both chemicals altered expression in some genes in a similar fashion, each induced unique gene expression patterns.

The primary goal of this study was to look for consistent changes common to all donors that could potentially be used as biomarkers of exposure, creating a gene expression profile following OTQ exposure in normal human cells. Given previous research in hepatoxicity, ideally normal human liver cells would have been used as a model system. However, we needed a normal human tissue that was readily accessible. Therefore, gene expression was studied in primary normal human mammary epithelial cells from four different donors in response to OTQ exposure. Current microarray analysis of pesticide exposure focuses on animal studies, not allowing for analysis of inter-individual variation. With the use of microarrays including clinical diagnosis, genetic susceptibility to disease and treatment, the need to determine the potential role of inter-individual variation on gene expression patterns in all potentially exposed tissues. Genes found to be altered in multiple cell strains could be considered biomarkers for individual populations. Given the small number of cell strains used, follow-up analysis in a larger number of samples is required to confirm any potential biomarkers. Biomarkers derived from this study could potentially be used in future epidemiology studies analyzing the effects of pesticide exposure. Further, this gene expression profile could be compared to those of other pesticides and of known carcinogens to develop a more detailed mechanistic definition of these chemicals.

## Methods

### Cell culture

Primary normal human mammary epithelial cells (NHMECs) were derived from tissues salvaged at reduction mammoplasty obtained though the Cooperative Human Tissue Network (National Cancer Institute and National Disease Research Interchange). Development and characterization of cell strains was achieved using standard methods [21]. Cells were grown in MEGM media (Clonetics, Cambrex, Pittsburgh, PA) at 37°C and 5% CO_2_.

### OTQ treatment

Treatment was performed on cells in passage six at 70% confluency, as routinely performed in our laboratory. Preliminary studies analyzed a range of OTQ concentrations (0 – 12.5 μM) selected based on previous research [6,7] and time points (0 – 24 hours), and showed maximum effect of OTQ on *p53 *expression with minimal toxicity at 2 hours with a final concentration of 6.25 μM. Cells were treated by diluting the stock OTQ/DMSO mixture in media and adding this solution to aspirated cells, allowing even exposure to all cells. DMSO (0.001%) alone was used as a vehicle control. At the end of the treatment period, cells were removed for RNA isolation. Cell viability was determined by Trypan Blue exclusion assay.

### Indirect immunofluorescence

Confluent cells in passage five were trypsinized and plated on eight-well slides (LabTek II Slide System, Nunc, Naperville, IL). These cells were grown to 70% confluency before being treated with OTQ (6.25 μM) for 15, 60 and 120 min or DMSO (0.001%) for 120 min. At the end of treatment, the media was aspirated and the cells were fixed with methanol. Slides were then stained with anti-*p53 *antibody (1:1000, DO-1, Santa Cruz Biotech, Santa Cruz, CA) and incubated overnight at 4°C. The next day, the media was again removed and the secondary antibody (1:1500, goat anti-mouse FITC, Santa Cruz Biotech) was incubated for one hour at room temperature. Slides were washed in triplicate with phosphate buffered saline (PBS) and cover slips were added. Slides were dried for one hour at room temperature before viewing, using the laser scanning confocal microscope BX50 (Olympus), and quantitative analysis was performed [22]. Relative *p53 *expression was quantified between different time points and strains by determination of the area under the integrated intensity curve (Fluoview, Olympus, B & B Microscopes, Pittsburgh, PA).

### Microarray analysis

Microarray analysis was performed in duplicate using the HuGeneFL high-density oligonucleotide microarrays (Affymetrix™, Santa Clara, CA). Protocols from the Affymetrix Expression Analysis Technical Manual were followed

RNA was isolated from cells with Trizol (Gibco, Grand Island, NY), followed by purification with RNEasy Mini Kit (Qiagen, Valencia, CA). Spectrophotometer measurements were required to give a 260/280 ratio of 1.9–2.1 for use in microarray analysis. Double-stranded cDNA was then synthesized from total RNA (Superscript Choice System, Invitrogen, Carlsbad, CA). An *in vitro *transcription (IVT) reaction (Enzo, Farmingdale, NY) was then performed to produce biotin-labelled cRNA from the cDNA. Excess biotinylated dUTPs were removed by RNEasy Mini Kit before being fragmented and added to a hybridization cocktail, including Eukaryotic Hybridization controls (Affymetrix), BSA and herring sperm DNA (Gibco, Grand Island, NY) and biotinylated anti-streptavidin antibody (Vector Laboratories, Burlingame, CA). Hybridization on microarrays was performed for 16 hours at 45°C in the Gene Chip Hybridization Oven with rocker (Affymetrix).

Microarrays were washed and stained using the protocol, as described in the Affymetrix Manual, with the GeneChip Fluidics Station 400 (Affymetrix). Arrays were then scanned with the Affymetrix Scanner (Hewlett Packard, Palo Alto, CA). Expression profiles were analyzed using Microarray Suite 5.0, MicroDB 3.0 and Data Mining Tool 3.0 (Affymetrix). Affymetrix arrays are produced using multiple 25-mer oligonucleotides (11–20 per target gene). Each oligonucleotide is created to match the selected region of the target gene (perfect match, PM), while a similar oligonucleotide is created altered in the 13^th ^position to control for non-specific binding (mismatch, MM). Results are given in signal intensities with a *p*-value determined from perfect match/mismatch (PM/MM) intensities by Tukey's Biweight analysis. Each array was normalized to a scaling factor of 1500 to correct for array variation. All arrays for each cell strain were analyzed on the same day to minimize variation.

Signal log ratio was determined by comparison of the signal intensities for the baseline (vehicle control) and the treatment array. This is computed using a one-step Tukey's Biweight method by taking a mean of the log ratios of probe pair intensities across the two arrays. This method helps to filter out differences due to different probe binding coefficients that may lead to false positives and/or negatives. A signal log ratio of zero represents no change in gene expression as a result of OTQ exposure. A signal log ratio of one is equivalent to a fold change of two between the treatment and control. The results described here are the average of both duplicates, with the average percent variability between duplicate arrays being 1.5% (the average difference found between duplicates, related to array to array variability as well as technical variability in processing the array). Only relative changes equal to or greater than 0.6 signal log ratio (SLR) were considered a significant change as a result of exposure. The biological significance of each change is determined with Wilcoxon's signed rank test with the Affymetrix software. Gene chip analysis was performed by self-organizing map (SOM) clustering, focusing on genes with a detection *p *value of 0.05 or less at one or more time points. Analysis was performed to comply with MIAME standards.

### Real-time polymerase chain reaction analysis (RT-PCR)

cDNA synthesized from each sample as in the Affymetrix analysis (Invitrogen) was used in a one-step RT-PCR analysis reaction. Analysis was performed in duplicate on the ABI 7700 cycler, with the SYBR Green Master Mix (ABI) and samples were normalized using both 18S and GAPDH expression levels for each sample. Primers were designed using Primer Express^® ^(ABI) to yield unique fragments for each gene under study. Reactions were set up following recommended protocols using 100 pmol of each primer (Sigma-Genosys) and approximately 60 ng template per reaction. Reactions were performed in duplicate for each sample for 40 cycles (95°C/15 sec denaturing step; 60°C/1 min annealing/extension step). Fold change was determined based on average cycle threshold (C_T_) values for all duplicates and converted to signal log ratio.

## Results

### Trypan blue exclusion test

Trypan Blue was used to analyze toxicity by measuring cell viability for each cell strain for each treatment. The results showed a range of viability from 92–97% at all time points, except for the last time point in strain 3, which had a viability of only 65% at 120 min (results not shown). This decrease in viability at 120 min was not found to be directly correlated to p53 or p21 protein expression, or to any particular gene expression pattern. The dose of OTQ used was based on these findings.

### Indirect immunofluorescence

**Figure 1:**
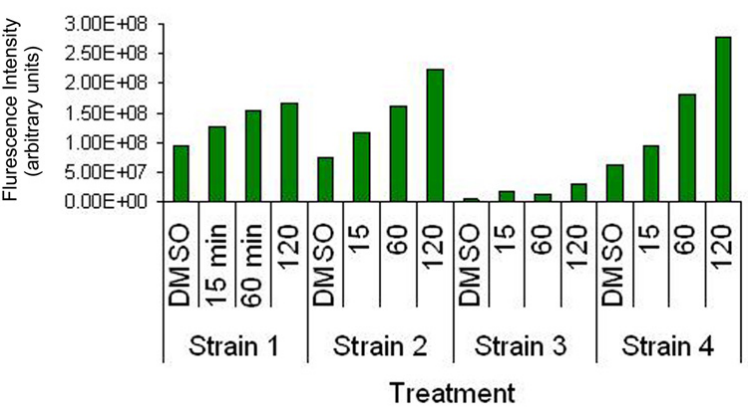
**Quantitative analysis of immunofluorescence microscopy. **Confocal microscopy analysis of *p53 *expression. Integrated intensity measures were obtained from Fluoview and graphed with GraphPad Prism (GraphPad Software, San Diego, CA) to determine area under the curve as a measure of comparative *p53 *protein expression for each treatment time point. Data is shown for each time point per cell strain on the ordinate and the intensity on the abscissa (arbitrary units, AU). There was an increase in *p53 *protein expression for all of the strains tested in direct correlation with OTQ exposure. In one intermediate strain (3), *p53 *expression was 10-fold lower at all time points.

Baseline *p53 *protein levels were visually compared to those after treatment in each cell strain. Integrated fluorescence intensities were measured on each optical slice of cells. The fluorescence was determined as the area under the curve in arbitrary units (AU) (Figure 1). This result was compared between time points for each cell strain. An increase was seen in *p53 *expression in all cell strains with increasing duration of an OTQ exposure. However, one strain (3) showed a 10-fold lower *p53 *expression at each time point.

### DNA microarray

DNA microarray analysis found no change in *p53*, despite the increase in *p53 *protein levels observed by immunofluorescence. Studies of benzo [a]pyrene exposure in our laboratory have also found similar results for *p53 *expression [23]. The effect of OTQ exposure on other cell cycle genes, however, was determined by DNA microarray analysis.

Although inter-individual variation between donors in response to OTQ was evident, there were also some genes found to be increased consistently in all strains by microarray analysis (Table 1). Self-organizing map (SOM) clustering was used to group genes with similar patterns of alteration in each of the strains. SOM analysis was performed following filtering of the total genes on the array, limiting the SOM analysis to only genes found to be present on at least one array analyzed. Following suggested analysis with the Affymetrix system, the default settings of the Affymetrix software were selected, including selecting threshold filtering (min = 20, max = 20000), row variation filtering (max/min = 3 and max-min = 100), and row normalization (mean = 0, variance = 1). Working with a 3 × 3 analysis to obtain 9 clusters generally gave an optimal amount of different clusters with less than 100 genes per cluster. From the SOM clustering analysis, genes altered by a signal log ratio of ± 0.6 or greater were chosen for closer study.

**Table 1:** Genes altered following oxythioquinox exposure. Table represents data mined from HuGeneFL microarrays (Affymetrix). All genes selected have a signal log ratio of ± 0.6 unless otherwise noted. Representative genes for each group were selected based on their function and are shown here.

GenBank ID	Name	Peak Expression Level (SLR)	Functional Class
Genes increased in three or more strains (n = 13):			
U22028	CYP2A13	1.5	xenobiotic metabolism
U20734	junB	3.42	transcription
V01512	cfos	2.04	transcription
S85655	prohibitin	0.75	cell proliferation
S82240	RhoE GTPase	2.81	signal transduction
M69043	MAD-3 mRNA encoding IkB-like activity	0.83	apoptosis
M63573	cyclophilin	1.68	immune response
U05861	Dihydrodiol dehydrogenase	2.52	xenobiotic metabolism
Genes decreased in three or more strains (n = 23):			
L05624	MAP Kinase Kinase	-0.67	signal transduction
U18018	E1A enhancer	-1.34	transcription
X56681	junD	-1.03	transcription
X68836	S-adenosylmethionine synthetase	-2.57	cell metabolism
J04973	Cytochrome bc-1	-3.12	cell metabolism
Genes altered in at least two of four cell strains (n = 189):			
X03484	raf oncogene	1.46	carcinogenesis
M60974	growth arrest and DNA-damage-inducible protein (gadd45)	1.51	DNA damage
M57731	Human gro-beta	1.85	immune response
X66899	EWS	1.2	carcinogenesis
Z29087*	Cyclin D1 Promoter	1.03	cell cycle control
L10910	Splicing Factor CC1.3	0.62	RNA processing
M83667	NF-IL6 Permeability Factor	1.55	transcription
M27281	Vascular Permeability Factor	-1.09	cell proliferation
L28010	HnRNP F protein	-0.55	RNA processing
U72649	BTF2	-1.86	carcinogenesis
M19267	tropomyosin	-1.16	cardiac
M38258	retinoic acid receptor gamma 1	-0.94	cell metabolism
U42031	Immunophilin	-1.41	immune response
U67122	ubiquitin-related SUMO-1	-1.03	protein metabolism
X70340	Transforming growth factor alpha	-0.57	cell proliferation
M34458	Lamin B	-1.33	cell proliferation

*No accession number was used by Affymetrix. This accession number most closely matches the probe description and sequence.

**Figure 2:**
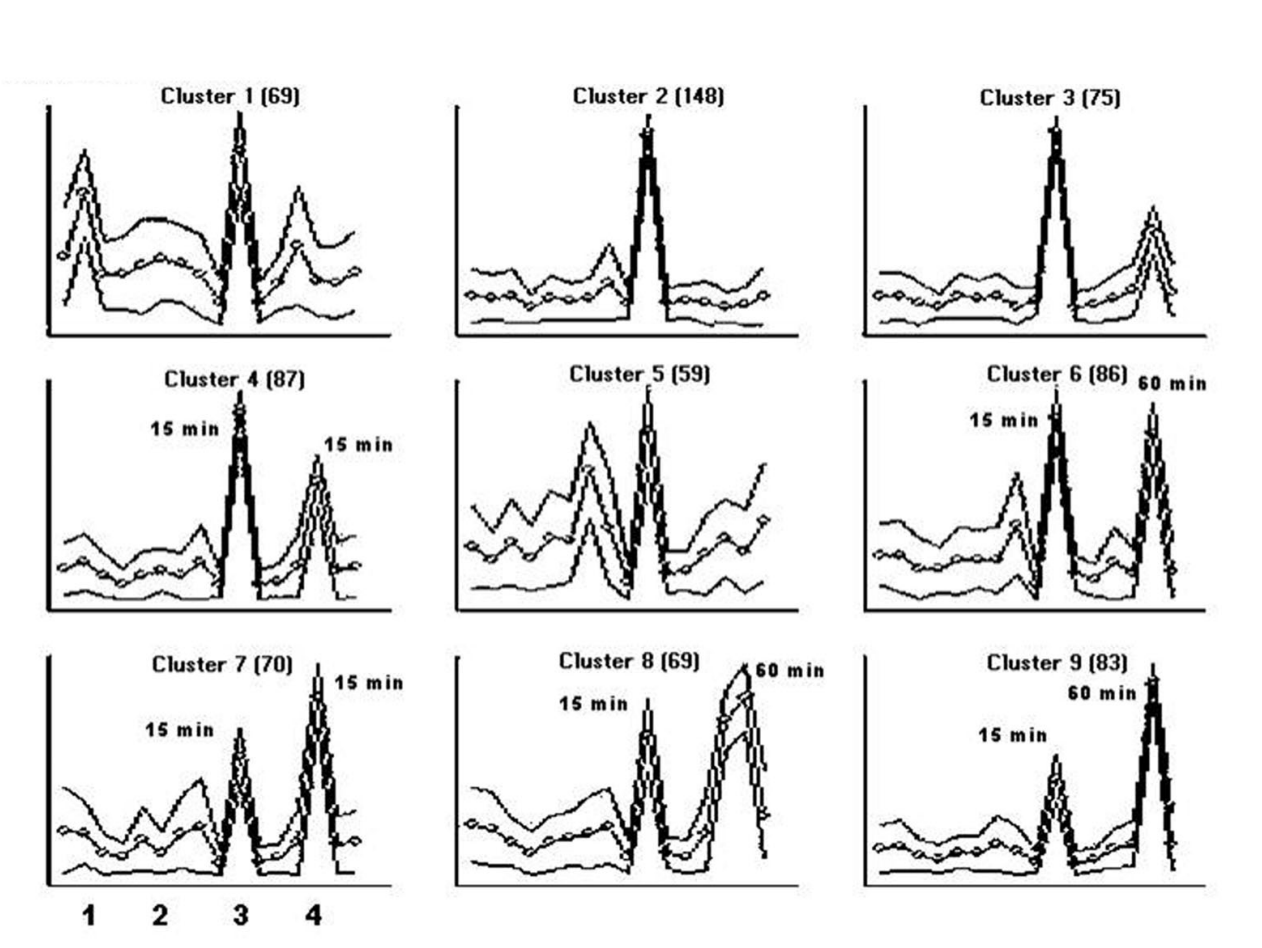
**SOM clustering. **Self-organizing map clustering groups' genes by similar expression patterns. Each data point represents an OTQ treatment time point. Data points are plotted in order of cell strain with four points per strain (DMSO, 15 min, 60 min, and 120 min). Levels of expression are not given, as patterns of expression are based on relative expression levels. Panels 4 and 7 of this graph show genes increased at 15 minutes for one strain (3) follow similar pattern in second strain (4). Similar pattern is also seen in panels 6 and 8 of this graph.

SOM clustering was used to show patterns of expression in each cell strain, and followed by further subclustering of those clusters of interest across all cell strains (Figure 2). This analysis was performed with Data Mining Tool 3.0 (Affymetrix). For comparison to earlier versions of the Affymetrix software, a signal log ratio of one is equal to a fold change of two. SOM clustering led to the selection of genes found to be altered, with the overall total of 215 genes (13 increased in 3 or more strains; 23 decreased in 3 or more strains). To be included, the genes must have a signal log ratio of ± 0.6, which is approximately a 1.5 fold change. Signal log ratio was used to linearize the data for ease of analysis. The full list of genes altered can be found at .

**Figure 3:**
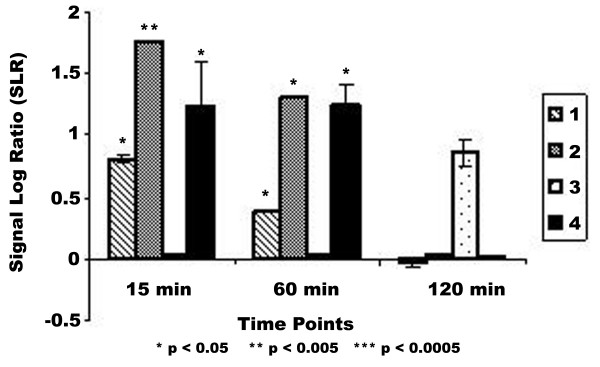
**Expression pattern for CYP2A13. **DNA microarray analysis of NHMEC strains. Analysis was performed as described on HuGeneFL high-density oligonucleotide microarrays (Affymetrix). Results are plotted as duration of exposure vs signal log ratio (SLR). Signal log ratio is a measure of comparative expression of the treatment vs. vehicle control (0.001% DMSO). Signal log ratio of one is equal to a fold change of two. A SLR of 0.6 (Fold Change ~1.5) was the arbitrary limit of our analysis. All genes given an Absent call by analysis software are shown with a SLR of zero. Asterisks indicate a statistically significant variation in expression from the control level as measured by Tukey's Biweight analysis.

**Figure 4:**
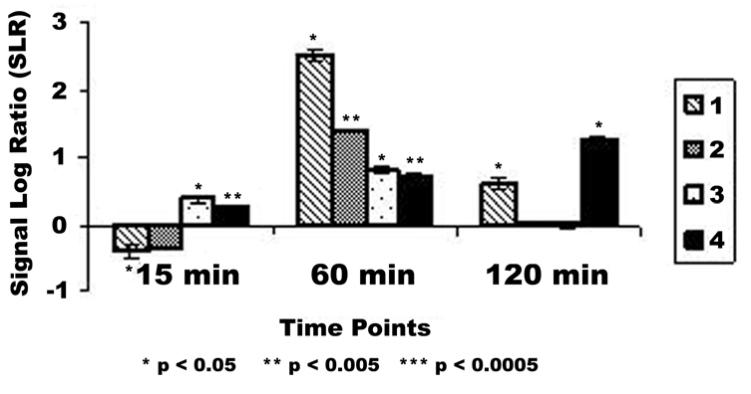
**Expression pattern for dihydrodiol dehydrogenase. **DNA Microarray analysis of NHMEC strains. Analysis was performed as described on HuGeneFL high-density oligonucleotide microarrays (Affymetrix). Results are plotted as duration of exposure vs signal log ratio (SLR). Signal log ratio is a measure of comparative expression of the treatment vs. vehicle control (0.001% DMSO). Signal log ratio of one is equal to a fold change of two. A SLR of 0.6 (Fold Change ~1.5) was the arbitrary limit of our analysis. All genes given an Absent call by analysis software are shown with a SLR of zero. Asterisks indicate a statistically significant variation in expression from the control level as measured by Tukey's Biweight analysis.

From the full list of genes that fit these criteria, selected genes were chosen due to their potential role in carcinogenesis, whether by cell cycle control, immune response or other specific functions. Functions were described as annotated by NetAffx [24]. These genes are listed in Table 1. Some genes were selected based on their possible role in disease as a result of pesticide exposure. Table 1 contains 8 genes increased in at least three of the four cell strains analyzed by 1.5 fold, as compared to the vehicle control and a list of 5 genes decreased in three or more of the four strains analyzed. Included in this list are two genes involved in the metabolic activation of endogeneous chemicals, *cytochrome P4502A13 (CYP2A13) *and *dihydrodiol dehydrogenase*. Although expression was slightly increased at one time point, the temporal patterns were slightly different (Figures 3 and 4). This increase in all cell strains suggests a potential role for these genes in OTQ metabolism. These results are confirmed at these time points as well as at 12 h and 24 h by RT-PCR (Table 2). Genes also found to be increased in at least three of four cell strains analyzed include genes involved in transcription (*junB*, *cfos*), immune response (*cyclophilin*), and apoptosis (*MAD-3*). These genes showed a consistent increase in expression following exposure to OTQ (Table 1). Genes that showed a consistent decrease in expression following exposure include signalling pathway genes (*MAP kinase kinase*), cell metabolism genes (*S-adenosylmethionine synthetase*, *cytochrome bc-1*), and transcription factors (*E1A enhancer*).

**Table 2:** RT-PCR results. Table represents real-time PCR data from selected genes of interest. Time points used were 15 min, 120 min, 12 h and 24 h where shown. Results shown are results of replicate analysis with duplicate samples. Samples not analyzed represented by N/A.

Strain	Gene	SLR 15 min	SLR 120 min	SLR 12 h	SLR 24 h
1	BTF2	1.41	N/A	1.53	0.3
2	BTF2	-1.56	N/A	-1.32	-1.74
3	BTF2	0.18	N/A	-0.89	-16.61
4	BTF2	0.62	N/A	-0.36	0.65
1	CYP2A13	5.58	6.01	3.84	10.6
2	CYP2A13	2.7	0.62	1.1	-0.6
3	CYP2A13	5.76	4.77	-0.6	-1.64
4	CYP2A13	5.45	1.1	0.33	-1.51
1	DDH	1.23	-0.67	1.75	1.47
2	DDH	-0.47	-0.62	0.96	5.41
3	DDH	0.86	-3.47	2.01	-3.84
4	DDH	3.44	N/A	5.53	6.09
1	EWS	-1.18	-1.84	0.69	-5.64
2	EWS	-6.64	-0.4	1.32	1.54
3	EWS	-0.42	-0.22	-1.79	-6.64
4	EWS	N/A	2.92	0.78	0.86
1	GADD45	-0.09	-0.22	-1.32	-5.06
2	GADD45	-1.03	-1.69	-1.94	-1.32
3	GADD45	0.82	-2.32	-1.56	-4.32
4	GADD45	1.34	9.05	6.4	7.72
1	MAD-3	-0.71	N/A	-0.15	-3.64
2	MAD-3	-1.89	N/A	-1.06	-0.38
3	MAD-3	0.34	N/A	-0.58	-6.64
4	MAD-3	4.4	N/A	-4.38	0.51
1	PROHIBITIN	0.31	N/A	-0.3	0.96
2	PROHIBITIN	-0.6	N/A	0.3	-1.18
3	PROHIBITIN	-1.06	N/A	-1.94	-2.84
4	PROHIBITIN	2.3	N/A	-1.09	0.92
1	RAF	0.01	-2.25	1.21	-5.64
2	RAF	-1.51	-0.27	-1.43	-0.06
3	RAF	0.37	-3.84	-1.12	-7.67
4	RAF	1.33	7.57	4.8	7.22
1	RhoE	0.59	N/A	-0.62	-0.71
2	RhoE	0.26	N/A	-1.18	1.08
3	RhoE	1.55	N/A	-0.94	-5.64
4	RhoE	2.35	N/A	1.06	2.05

Some genes of interest were variably altered by strain, examples of which are also listed in Table 1. These include genes involved in carcinogenesis (*raf oncogene*, *GADD45*, *EWS*, *Cyclin D1*) as well as immune response (*immunophilin*, *gro-β*), cell proliferation (*TGFβ*), and RNA processing (*HnRNP F protein*).

### Real-time PCR

Real-time PCR was used to confirm and extend results seen by microarray analysis for selected genes. Following the original microarray analysis, patterns of some genes appeared to be changing at the latest time point (120 min), so extended time points were selected (12 and 24 h) to see a more complete expression profile for these genes. Due to limited amount of cDNA, genes were analyzed at 15 min and/or 120 min, and then analyzed at 12 and 24 h by RT-PCR. Extended time points were selected to look at specific genes found altered at the earlier time points. These genes were selected due to their function and/or pattern of expression, and determining their expression pattern at later time points was of interest. Results are shown in Table 2. In the majority of samples, RT-PCR confirmed data found by microarray analysis for the genes listed. Some discrepancies are also shown, however, in these cases it is believed that the primer sequence is more specific to the gene in question than the probe sequence used on the array. However, extended time points, in some cases, showed that the results of early time points did not always continue to extended exposures. The RT-PCR results for *prohibitin*, *DDH*, and *CYP2A13 *at 24 h showed a decrease in expression in some of the cell strains analyzed. Samples not available for RT-PCR analysis are listed as N/A in Table 2.

## Discussion

The purpose of this study was to determine if microarray analysis of four normal human mammary cell strains with a known haplotype could be used to find biomarkers related to either exposure in general or the specific haplotype in question. The use of only four cell strains was determined to have enough power to provide basic information to lead to further study if necessary. This study would be followed up for specific genes of interest in a larger number of cell strains, preferably bypassing the more expensive and time-consuming microarray analysis for RT-PCR only.

DNA microarray analysis was used to profile the cellular response to OTQ, a quinoxaline pesticide. Analysis revealed genes with common response across the four human cell strains studied as well as inter-individual variation in response. Using only four normal human cell strains, our goal was to discover any distinctly altered genes in response to OTQ exposure. Future analysis with a larger number of cell strains will be used to follow-up this analysis on specific genes of interest.

The majority of studies looking at gene expression profiles have used animal models, limiting any knowledge obtained to genetically similar organisms. Analysis with normal human cell strains, like those described here, will give more information on inter-individual variation in response to various chemicals. This information will yield clues to the metabolic pathways of the specific chemicals, and this increased knowledge will aid in determining potential hazards in the environment and the workplace. Given the large number of pesticides in use today, further examination of the effect of these chemicals on individuals is warranted.

Following exposure to OTQ, NHMECs showed alterations in genes involved in a variety of functions. These included xenobiotic metabolism, transcription, and DNA synthesis. Genes altered as a result of OTQ exposure in all strains analyzed included transcription factors like *junB *and *cfos *(the AP1 complex). A number of genes involved in carcinogenesis were found altered after exposure to OTQ, both induced and down-regulated. For example, *prohibitin *expression is found to be up-regulated in most cell strains after OTQ exposure, with similar expression patterns associated with a decrease in cancer incidence [25].

Metabolism genes that were altered after OTQ exposure, like *cytochome P4502A13 (CYP2A13) *and *dihydrodiol dehydrogenase*, are involved in xenobiotic metabolism. It has been suggested that CYP2A13 is the main metabolic activator of 4-(methylnitrosamino)-1-(3-pyridyl)-1-butanone (NNK), a tobacco-specific nitrosamine [26]. The increase of *CYP2A13 *begins to wane at the final time point tested, suggesting this alteration to be somewhat transient. This is an unusual expression pattern for a cytochrome p450, as genes in this family tend to show a gradual increase in induction, and an equally gradual decrease in expression. Further analysis by RT-PCR showed that this p450 was increased at later time points (12 h and 24 h, Table 2). Another metabolic enzyme affected by OTQ exposure is dihydrodiol dehydrogenase. Dihydrodiol dehydrogenase is known to participate in activation of certain polycyclic aromatic hydrocarbons (PAHs), so its increase in the intermediate variant after OTQ exposure may result in an increase in PAH activation [27,28]. Alterations of genes like this may suggest an indirect role for OTQ in carcinogenesis. Of these two genes, only CYP2A13 seems specific to exposure to OTQ. DDH has been found to be increased following exposure to various chemicals, including malathion, di-n-butyl phthalate, and benzo [a]pyrene (Gwinn in preparation, 2004) [23,29].

Some of the early results at 15 min by microarray analysis may have been a consequence of the stress of exposure, regardless of the chemical. Extending analysis to later times determined whether results seen at these early time points were still valid after longer exposure to OTQ. RT-PCR results given in Table 2 show that in most cases, the extended time points showed a continued trend of expression (whether increased or decreased), except in some cell strains for *DDH*, *prohibitin*, and *MAD-3*. These results show a reverse of the early expression patterns at the later time points. Real-time PCR is a more specific method of analysis, as it only interrogates one gene at a time with primers designed uniquely to that gene. Conflicts in results between the two methods can generally be attributed to cross-reaction between probes designed for similar genes on the array. Sequence differences between the probes on the array and those used in RT-PCR may also play role in these results. The RT-PCR primers were selected specifically for the gene in question, while the probes on the array may not have been. Genes with sequence homology but with altered patterns of expression may not have been differentiated in the array analysis, but would be with the RT-PCR analysis.

Inter-individual variation as a result of genetic polymorphisms in genes of interest would focus on specific at-risk worker populations. For example, the four cell strains analyzed in this study have been genotyped for a variety of genes, in particular those involved in cell cycle control and xenobiotic metabolism. Two of the four cell strains selected for analysis are heterozygote for the minor variant haplotype of p53, a cell cycle control gene (cell strains 3 and 4). Although no biological mechanism for the role of this variant in carcinogenesis has been defined, several studies associating this haplotype with various cancers support such role [30-35]. Analysis of genes altered in just those strains expressing this variant, including three genes involved in cell cycle control: *raf oncogene *(X03484), *cyclin D1 *(Z29087), and *BTF2 *(U72649) may further support an association between OTQ exposure, p53 variant status and carcinogenesis [36-38]. Of these, p53 has been reported to increase *GADD45 *transcription in response to DNA damage, which is associated with an increase in cell cycle arrest and DNA damage repair, while increased levels of *raf oncogene *have been associated with lung carcinogenesis [39-41].

Given the small number of cell strains used, this analysis needs to be extended to additional cell strains to determine the role of the *p53 *variants in gene expression differences. Due to the expense of microarray analysis, this is performed in only a limited number of cell strains (4), and selected genes will be further analyzed with |RT-PCR in a larger number of cell strains. Over 80 cell strains have been established in our laboratory to date, with half of these having been genotyped for *p53*. However, given that this haplotype is found only in a limited portion of the population, this varied pattern of expression in key genes in cell cycle control may highlight a specific at-risk population.

Searches for similar natural compounds to replace these potentially disruptive chemicals can also use gene expression profiles [42]. Profile comparisons to that of a natural pesticide may decrease the need for organophosphates. Comparison of OTQ's gene expression profile to that of well-defined chemicals, like benzo [a]pyrene, will yield important information about OTQ's role in both genotoxicity and potential carcinogenicity. A comparison between many expression profiles is needed to further define similarities and differences between this pesticide and known carcinogens and/or other pesticides.

## Conclusions

The overall goal of this project was to create a gene expression profile for OTQ or related pesticide analogues with the hopes of finding genes to be used as potential biomarkers of exposure. This expression profile may also be used to determine the final role of OTQ in carcinogenesis by comparing it to profiles of known carcinogens. It is possible that the main effect of OTQ exposure is not on the direct alterations in many genes, but on alterations in genes potentially involved in carcinogenesis, among them the examples of *CYP2A13 *and *dihydrodiol dehydrogenase*. The results shown here do not suggest a direct role of OTQ in carcinogenesis. They do, however, suggest OTQ exposure leads to an increase in expression of genes that do play a direct role in carcinogen; metabolism (for example, exposure to carcinogens like NNK and benzo [a]pyrene along with exposure to OTQ may lead to an increased incidence of tobacco-related cancer) [27,43].

Discovery of genes altered following exposure to OTQ in human cell strains may aid in future epidemiology studies on pesticide exposures. Gene expression profiling can be used to yield genetic biomarkers of exposure that, after validation, could be used in a clinical setting for early determination of organophosphate exposure, increasing early treatment of pesticide illness and thereby increasing the recovery rate of exposed individuals.

## List of abbreviations used

OTQ, oxythioquinox

DDH, dihydrodiol dehydrogenase

DMSO, dimethylsulfoxide

SLR, signal log ratio

DMT, Data Mining Tool™

MAS, Microarray Suite™

SOM, self-organizing map

NHMEC, normal human mammary epithelial cell

RT-PCR, real-time polymerase chain reaction

## Competing interests

None declared.

## Authors' contributions

MRG participated in the design of the study, and performed all experiments. DLW was responsible for the growth and maintenance of all cell strains used. AW conceived of the study and participated in the design and coordination. All authors read and approved the final manuscript.

## Pre-publication history

The pre-publication history for this paper can be accessed here:


